# Acute Responses to Oxygen Delivery via High Flow Nasal Cannula in Patients with Severe Chronic Obstructive Pulmonary Disease—HFNC and Severe COPD

**DOI:** 10.3390/jcm10091814

**Published:** 2021-04-21

**Authors:** Amy H. Attaway, Jihane Faress, Frank Jacono, Srinivasan Dasarathy

**Affiliations:** 1 Department of Pulmonary, Allergy and Critical Care Medicine, Respiratory Institute, Cleveland Clinic, 9500 Euclid Avenue, NE4 208, Cleveland, OH 44195, USA; attawaa@ccf.org; 2 Division of Pulmonary, Critical Care and Sleep Medicine, Louis Stokes Cleveland VA Medical Center, Cleveland, OH 44106, USA; jihane.faress1@uhhospitals.org (J.F.); frankjacono@gmail.com (F.J.); 3 Department of Inflammation and Immunity, Lerner Research Institute, Cleveland Clinic, 9500 Euclid Avenue, NE4 208, Cleveland, OH 44195, USA

**Keywords:** high flow nasal cannula, COPD, hypercapnia, chronic respiratory failure, respiratory, oxygen inhalation therapy, ventilation perfusion mismatch, structural lung disease

## Abstract

Differences in oxygen delivery methods to treat hypoxemia have the potential to worsen CO_2_ retention in chronic obstructive lung disease (COPD). Oxygen administration using high flow nasal cannula (HFNC) has multiple physiological benefits in treating respiratory failure including reductions in PaCO_2_ in a flow-dependent manner. We hypothesized that patients with COPD would develop worsening hypercapnia if oxygen fraction was increased without increasing flow rate. We evaluated the acute response to HFNC in subjects with severe COPD when flow remained constant and inspired oxygen was increased. In total, 11 subjects with severe COPD (FEV1 < 50%) on supplemental oxygen with baseline normocapnia (PaCO_2_ < 45 mm Hg; *n* = 5) and hypercapnia (PaCO_2_ ≥ 45 mm Hg; *n* = 6) were studied. Arterial blood gas responses were studied at three timepoints: Baseline, HFNC at a flow rate of 30 L/min at resting oxygen supplementation for 1 h, and FiO_2_ 30% above baseline with the same flow rate for the next hour. The primary endpoint was the change in PaCO_2_ from baseline. No significant changes in PaCO_2_ were noted in response to HFNC applied at baseline FiO_2_ in the normocapnic and hypercapnic group. At HFNC with FiO_2_ 30% above baseline, the normocapnic group did not show a change in PaCO_2_ (baseline: 38.9 ± 1.8 mm Hg; HFNC at higher FiO_2_: 38.8 ± 3.1 mm Hg; *p* = 0.93), but the hypercapnic group demonstrated significant increase in PaCO_2_ (baseline: 58.2 ± 9.3 mm Hg; HFNC at higher FiO_2_: 63.3 ± 10.9 mm Hg; *p* = 0.025). We observed worsening hypercapnia in severe COPD patients and baseline hypercapnia who received increased oxygen fraction when flow remained constant. These data show the need for careful titration of oxygen therapy in COPD patients, particularly those with baseline hypercapnia when flow rate is unchanged.

## 1. Introduction

High flow nasal cannula (HFNC) is a potent adjunctive therapy for patients with acute hypoxemic respiratory failure in the ICU [1,2]. The heated humidifier system improves mucociliary function and decreases the metabolic cost of gas conditioning while the flow enhances oxygen delivery, generates a low-level positive airway pressure, and reduces respiratory rate [3,4,5,6,7,8]. HFNC has emerged as an alternative to and less invasive treatment for respiratory failure than noninvasive positive pressure (NIPPV) or invasive mechanical ventilation (IMV) [9]. Recent randomized control trials have reported decreased mortality at 90 days of HFNC compared to NIPPV and conventional oxygen in the treatment of acute hypoxemic respiratory failure [1] and reduced rates of reintubation at 72 h when compared to conventional oxygen devices or NIPPV [2,10]. Moreover, HFNC use for acute hypoxemic respiratory failure during the COVID-19 pandemic [11,12,13,14,15,16,17,18,19] was associated with successful outcomes 34–70% of the time. Computer modeling has suggested that incorporating HFNC as a strategy for patients could result in greater mechanical ventilator availability and potentially fewer deaths [18] during the COVID-19 pandemic.

**Figure 1:**
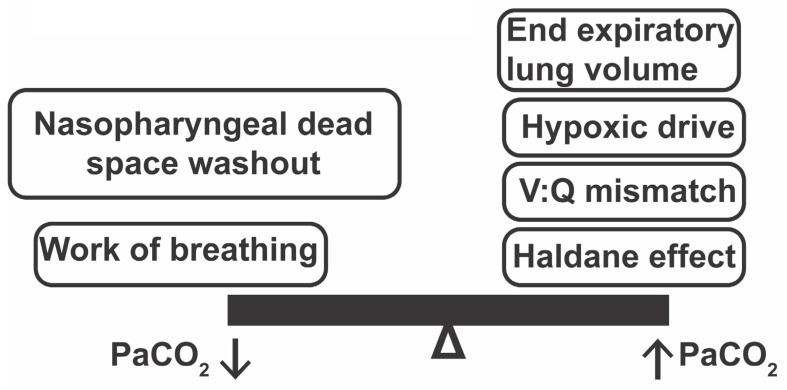
Physiologic mechanisms that may improve or worsen hypercapnia when implementing HFNC in a patient with structural lung disease like COPD. V:Q mismatch = Ventilation : Perfusion mismatch.

In patients with COPD, previous studies have reported overall reductions in end tidal CO_2_ in COPD patients in a flow-dependent manner [5] or reductions in PaCO_2_ in response to HFNC [20,21,22,23,24,25]. However, the response of individual patients can vary. As shown by Nilius et al., of 17 patients with COPD who received HFNC, 7 patients showed increased PaCO_2_ (with 3 increasing > 4 mm Hg), 9 showed decreased PaCO_2_, and 1 remained unchanged [26]. The category of patients who develop worsening hypercapnia with oxygen therapy is relevant due to their risk for cardiopulmonary decompensation and worsened clinical outcome. Interestingly, studies have shown that HFNC is associated with decreased respiratory rate, but that PaCO_2_ often remains unchanged or decreased possibly due to improved ventilation [7,27,28] or reductions in respiratory effort and work of breathing leading to less PaCO_2_ production [27,28] (Figure 1) [5,6,7,29,30]. However, for patients with structural lung disease including COPD, increased oxygen fraction leads to oxygen-induced hypercapnia due to changes in hypoxic drive, the Haldane effect, and worsening ventilation/perfusion (V/Q) mismatch [31,32]. Administering 100% oxygen to patients with COPD has been reported to increase PaCO_2_, on average, by 23 mm Hg, with hypoxic drive responsible for an average of 5 mm Hg and V/Q mismatch responsible for 11 mm Hg [31,32]. It is also possible that increases in end expiratory lung volumes (a known consequence of HFNC) [5] may cause dynamic hyperinflation in patients with COPD and worsen PaCO_2_ retention [33]. Therefore, it is important to study the effects of both flow rate and inspired oxygen fraction in patients with severe COPD to better understand the multiple HFNC-related physiologic effects in these patients. We hypothesized that patients with COPD would develop worsening hypercapnia if oxygen fraction was increased without increasing flow rate. In patients with severe COPD and (FEV1 < 50%) on supplemental oxygen, we identified that patients with baseline hypercapnia were at risk for worsening hypercapnia when oxygen was delivered with HFNC when the oxygen fraction was increased by 30% but the flow rate remained unchanged.

## 2. Methods

From October 2016 to June of 2017, subjects were recruited from the pulmonary clinic at the VA Medical Center, Cleveland, Ohio. Written informed consent was obtained from all participants in accordance with the Helsinki declaration on ethical principles for medical research. Inclusion criteria for recruitment were severe COPD, defined as Global Initiative for Chronic Obstructive Lung Disease (GOLD) stage 3–4 criteria with FEV1/FVC < 0.70, FEV1 < 50%, and hypoxemic respiratory failure, defined as the need for supplemental oxygen at rest or with exertion. Patients with obstructive sleep apnea or obesity hypoventilation syndrome were excluded from the study, and all patients were low risk (<10) based on the Epworth sleepiness scale [34]. Patients with history of a neuromuscular disorder were also excluded. We also excluded those on anticoagulation or antiplatelets (other than daily aspirin 81 mg) and those who had a diagnosis of peripheral vascular disease, uncontrolled hypertension, end-stage renal disease, metastatic malignancy, or congestive heart failure (NYHA 3–4) [35]. Of the 206 patients who were screened, 54 qualified and 11 subjects agreed to participate. Patients were required to not be on long-term noninvasive ventilation at home and were clinically stable with greater than 6 weeks from their last COPD exacerbation. There were no missing data.

### 2.1. Design

**Figure 2:**
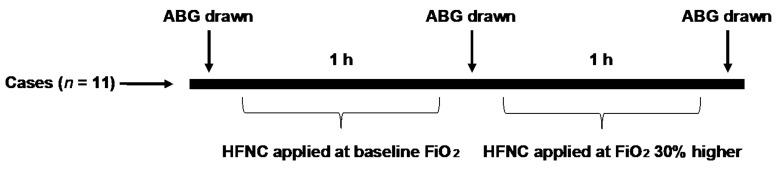
Schematic study design. After baseline ABG (arterial blood gas) was obtained on the patient’s supplemental oxygen (or room air if the patient used oxygen with exertion), subjects were placed on HFNC with an FiO_2_ equal to their baseline supplemental level and a flow rate of 30 L/min. After 1 h of clinical monitoring, a second ABG was drawn. FiO_2_ was then increased by 30% and the patient underwent a second hour of clinical monitoring, at the completion of which a third ABG was obtained.

A baseline arterial blood gas (ABG) was obtained using ultrasound guidance to minimize patient discomfort while patients were on their baseline supplemental oxygen delivered via standard nasal cannula. Subjects were then placed on HFNC with a FiO_2_ equal to their baseline supplemental level and a flow rate of 30 L/min. If the subject required oxygen only with exertion, then they were placed on an FiO_2_ of 21%. Respiratory rate and blood pressure were recorded every 20 min while pulse oximetry and transcutaneous capnography were recorded every 5 min. The subject answered a Borg dyspnea questionnaire [36] every 5 min. The results of the respiratory rate and Borg dyspnea questionnaire over the hour of treatment are presented as mean and standard deviation. At the completion of 1 h of HFNC, a repeat ABG was obtained, and FiO_2_ was increased to 30% above the subject’s baseline. We chose to increase the FiO_2_ by 30% because this is a significant increase above baseline that patients may encounter when they develop acute respiratory failure and require support with HFNC. If a research subject was receiving an FiO_2_ of 21% on initial HFNC, then the FiO_2_ was increased to 51% with the same flow rate of 30 L/min. Subjects underwent another hour of clinical monitoring as above, and at the completion of 1 h on the higher FiO_2_ setting, a third ABG was obtained (Figure 2). The study was terminated if PaCO_2_ was >70 mm Hg at initiation of the study, transcutaneous capnography increased by >30 mm Hg during the procedure, or a single reading was >80 mm Hg. At the completion of the study, subjects were monitored until they returned to their baseline CO_2_ level as measured by transcutaneous capnography. The HFNC device (Airvo2) was supplied by the company (Optiflow; Fisher & Paykel Healthcare, Auckland, New Zealand).

### 2.2. Sample Size and Statistical Analyses

Using a 2-tailed analysis and based on prior studies [4], we predicted the need for 5 subjects to have 90% power to detect a change in PaCO_2_ of 5 mm Hg, which is considered a clinically significant change [37]. Group data were assessed for normality and are either reported as mean ± standard deviation or median with interquartile range. Qualitative variables were compared using the chi-square test, and quantitative and rating variables were compared using paired t-tests. A *p*-value of less than 0.05 was considered significant. The primary outcome was change in PaCO_2_. Baseline normocapnia was defined as PaCO_2_ < 45 mm Hg, and baseline hypercapnia was defined as PaCO_2_ ≥ 45 mm Hg. Secondary outcomes for analysis included respiratory rate, change in PaO_2_, and Borg dyspnea score. Pearson’s correlation coefficient was calculated to compare transcutaneous CO_2_ to PaCO_2_ values as well as the change in respiratory rate and PaCO_2_ values. The R system 4.0.0 (R Foundation for Statistical Computing, Vienna, Austria) was used for statistical analysis. The studies were approved by the Institutional Review Board of the Louis Stokes Veterans Affairs Cleveland Medical Center and conformed to the Helsinki accord on human subjects in research (IRB# 16014-H08).

## 3. Results

Baseline characteristics for the study population are shown in Table 1. Patients were older, with a mean age of 66 years, and the median BMI was 25.50 kg/m^2^ (IQR 22.80, 33.55). Most patients (*n* = 9; 82%) were male, with an average FEV1 of 34% predicted. Average PaCO_2_ at baseline was 49.5 ± 11.9 mm Hg. The majority of subjects (*n* = 9; 82%) utilized continuous oxygen while the other two subjects used oxygen with exertion.

**Figure 3:**
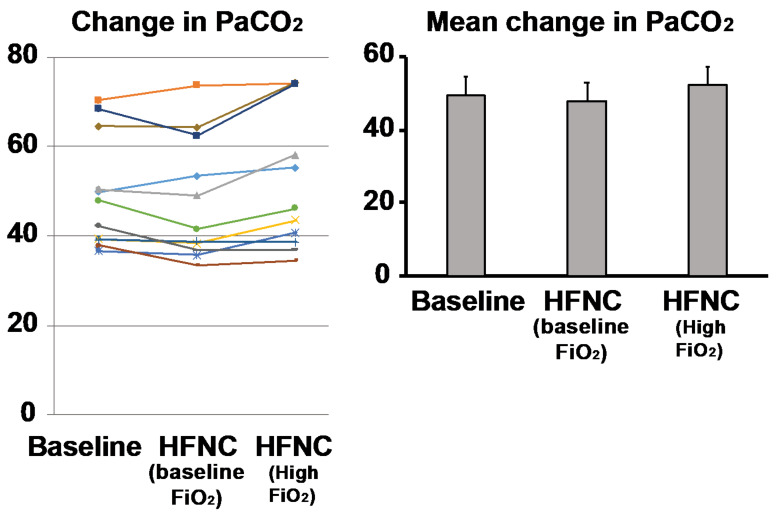
Response to baseline oxygen delivery via high flow nasal cannula (HFNC) in COPD patients. Comparison of baseline PaCO_2_ after being placed on HFNC at baseline FiO_2_ showed that the majority of subjects (*n* = 7; 63.6%) experienced a decrease in PaCO_2_, while others either stayed the same (*n* = 2, 18%) or developed increased PaCO_2_ (*n* = 2; 18%). After HFNC was increased by an FiO_2_ 30% above baseline there were increases in hypercapnia in the majority (*n* = 7, 63.6%). The baseline hypercapnic subjects (defined as resting PaCO_2_ > 45, *n* = 6) were more likely to increase their PaCO_2_ (*n* = 5, 83.3%). Each colored line represents the individual response of the patient.

Response to HFNC with baseline oxygen utilization was associated with a reduction in PaCO_2_ from 49.5 ± 13.1 mm Hg to 47.2 ± 14.1 mm Hg (*p* = 0.13). The normocapnic group (defined as PaCO_2_ < 45 mm Hg; *n* = 5) showed reductions in PaCO_2_ from 38.9 ± 1.8 mm Hg to 36.6 ± 1.9 mm Hg (*p* = 0.08). The hypercapnic group (*n* = 6) showed reductions in PaCO_2_ from 58.2 ± 9.3 mm Hg to 57.1 ± 10.5 mm Hg when HFNC was applied (*p* = 0.55) (Figure 3).

When HFNC was applied at 30% higher than baseline FiO_2_, PaCO_2_ increased from 49.5 ± 12.4 mm Hg to 52.1 ± 15.5 mm Hg (*p* = 0.09) compared to baseline in the entire cohort. In the normocapnic group, PaCO_2_ was unchanged (38.9 ± 1.8 mm Hg versus 38.8 ± 3.1 mm Hg; *p* = 0.93), while the hypercapnic group showed a significant increase in PaCO_2_ from 58.2 ± 9.3 mm Hg to 63.3 ± 10.9 mm Hg when FiO_2_ was increased (*p*= 0.03) (Figure 3).

Transcutaneous CO_2_ was monitored throughout and at the completion of the experiment. We found good agreement among the transcutaneous values and the PaCO_2_. Baseline transcutaneous CO_2_ was 48.0 ± 11.2 (R^2^ 0.97 compared to PaCO_2_, *p* < 0.001). After the completion of 1 h of HFNC at baseline FiO_2_, the value was 44.8 ± 10.6 (R^2^ 0.96 compared to PaCO_2_, *p* < 0.001), and after the completion of 1 h of HFNC at increased oxygen fraction, the value was 48.7 ± 12.9 (R^2^ 0.94 compared to PaCO_2_, *p* < 0.001).

**Figure 4:**
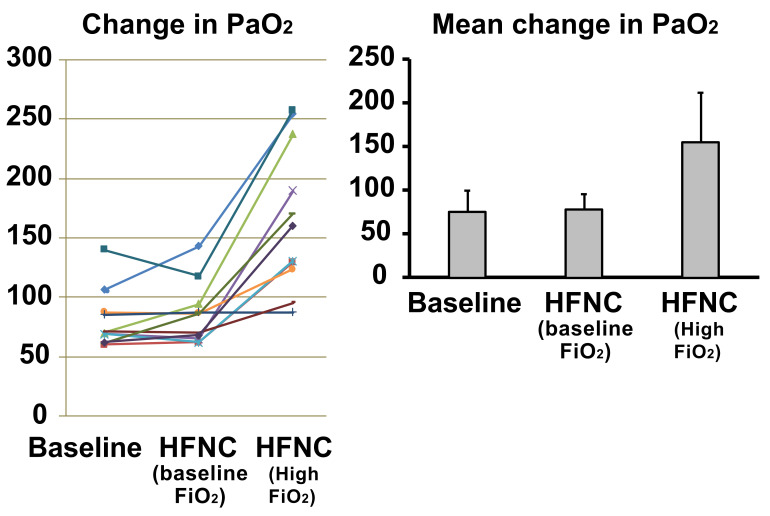
Response to increased oxygen delivery via high flow nasal cannula in COPD patients. The PaO_2_ did not show a significant change (*p* = 0.60) when HFNC was applied at baseline FiO_2_ and increased significantly (*p* < 0.001) when the FiO_2_ was increased by 30%. Each colored line represents the individual response of the patient.

**Figure 5:**
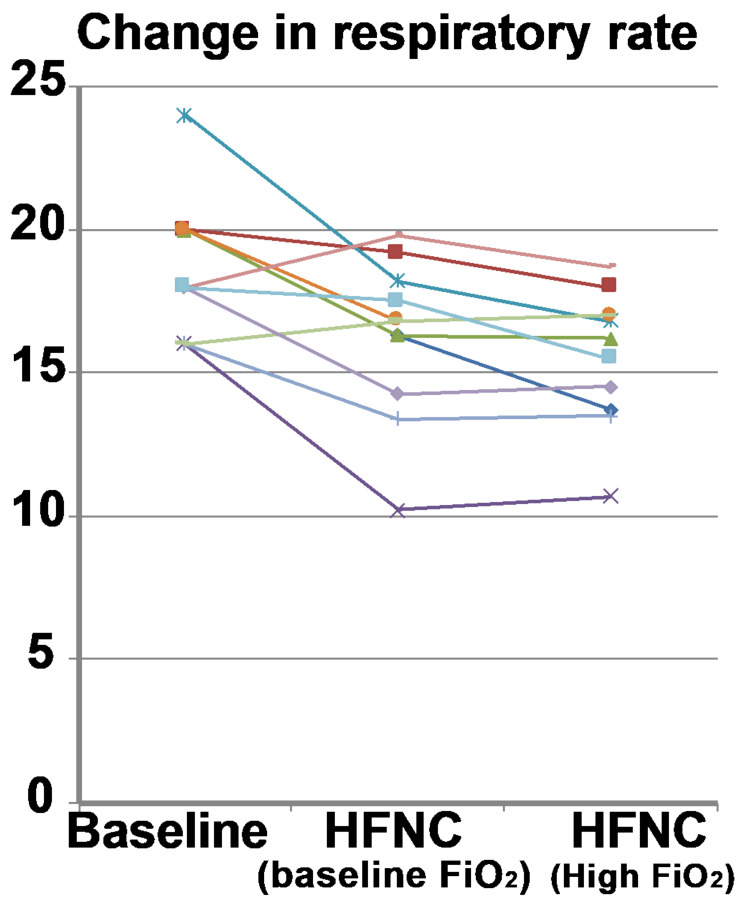
Baseline respiratory rate (respiratory rate) compared to high flow nasal cannula at baseline FiO_2_ and increased FiO_2_. The respiratory rate decreased in most subjects (*n* = 10; 90.9%) when HFNC was applied at baseline and at increased FiO_2_. Each colored line represents the individual response of the patient.

The PaO_2_ did not show a significant change (*p* = 0.60) when HFNC was applied at baseline (75.3 ± 23.5 mm Hg to 77.7 ± 17.6 mm Hg) and increased significantly (*p* < 0.001) when the FiO_2_ was increased by 30% (75.3 ± 23.5 mm Hg to 155 ± 56.0 mm Hg) (Figure 4). Baseline respiratory rate was 18.7 ± 2.4 breaths/min, which decreased to 16.3 ± 2.8 breaths/min after implementing HFNC at baseline FiO_2_ (*p* = 0.01). When the FiO_2_ was increased, the respiratory rate decreased from 18.7 ± 2.4 breaths/min to 15.6 ± 2.3 breaths/min (*p* < 0.001). There was no difference in respiratory rate when comparing HFNC at baseline FiO_2_ to increased FiO_2_ (*p* = 0.07) (Figure 5).

When comparing the respiratory rate between the normocapnic and hypercapnic subgroups, the normocapnic group did not show a significant change (baseline: 18 ± 3.1 breaths/min; HFNC: 15.7 ± 3.5 breaths/min; *p* = 0.22). However, the hypercapnic group showed a significant decrease in respiratory rate (baseline: 19.3 ± 0.94 breaths/min; HFNC: 16.7 ± 1.5 breaths/min; *p* = 0.01). At higher FiO_2_, the normocapnic group did not show a change in respiratory rate (baseline: 18.0 ± 3.1 breaths/min; HFNC at higher FiO_2_: 15.3 ±/− 2.9 breaths/min; *p* = 0.18), while the respiratory rate decreased significantly (*p* = 0.002) in the hypercapnic group (baseline: 19.3 ± 0.94 breaths/min; HFNC at higher FiO_2_: 15.8 ± 1.5 breaths/min; *p* = 0.002). Change in respiratory rate did not correlate significantly with the change in PaCO_2_ (R^2^ −0.32, *p* = 0.34). The Borg dyspnea questionnaire also showed no changes over baseline average score of 1.1 ± 1.3 compared to HFNC at baseline FiO_2_ (1.04 ± 1.2; *p* = 0.7) or higher FiO_2_ (0.98 ± 1.30; *p* = 0.61).

## 4. Discussion

Our studies in well-characterized patients with severe COPD showed that HFNC with increased inspired oxygen improved PaO_2_ but did not alter PaCO_2_ in normocapnic subjects. In contrast, in hypercapnic subjects, higher inspired oxygen delivered by HFNC with flow rate unchanged increased PaCO_2_ and lowered the respiratory rate. These observations suggest that when a higher FiO_2_ is delivered and flow remains the same, hypoxic drive is adversely affected, particularly in hypercapnic patients, and leads to worsening hypercapnia. We observed that HFNC led to reductions in PaCO_2_ at baseline oxygen fraction which approached statistical significance, which is consistent with published data that HFNC causes reductions in PaCO_2_ in a flow-dependent manner [5,20,25,38,39]. This was also observed in our hypercapnic cohort where the majority of patients developed reductions in PaCO_2_ compared to their baseline after initiation of HFNC. However, the unique aspect of our study was that when we increased the oxygen fraction at the same flow rate, this attenuated the improvements in PaCO_2_, particularly in the baseline hypercapnic population. This highlights the need to provide the least amount of oxygen to COPD patients and to augment the flow as patients tolerate to mitigate the risk of increased PaCO_2_.

HFNC is increasingly used for the treatment of acute hypoxemic respiratory failure as a less invasive mode of support than NIPPV and IMV, with studies demonstrating improvements in mortality and reintubation [1,2]. Its use is now being explored in domiciliary settings [22] for patients with COPD and chronic respiratory failure due to the positive physiologic benefits, including increased ventilation and reductions in work of breathing [7,8]. However, given the multiple physiologic factors at play, our study highlights the changes that occur when flow is kept constant but oxygen fraction is increased 30% above baseline. We chose to study 30% because this is a significant increase above baseline that patients may encounter when they develop acute hypoxemic respiratory failure and require support with HFNC. We therefore did not evaluate smaller increases in FiO_2_ (10% or 20%) because such increases are unlikely to recreate a clinical situation of acute respiratory failure requiring a significantly greater oxygen fraction than the patient’s baseline oxygen need. We recognize, however, that the lung mechanical conditions in our patients may be different than the conditions encountered during acute respiratory failure and that future studies in these clinical situations are needed. Whereas the decrease in respiratory rate from HFNC observed in our patients has been previously reported by others, in general, the PaCO_2_ does not increase when patients use HFNC [20,21,22,23,24,25]. This has been attributed to nasopharyngeal dead space wash out and the decreased work of breathing, which reduce PaCO_2_ levels [9]. However, the novel finding of our study is that COPD patients with chronic hypercapnic respiratory failure tended to increase PaCO_2_ when they received increased oxygen fraction via HFNC and the flow remained unchanged. In general, COPD patients with severe disease have a reduced ability to exhale carbon dioxide adequately, which leads to chronic elevation of PaCO_2_. The increase in carbon dioxide stimulates central and peripheral chemoreceptors to increase respiration, which are usually less responsive to oxygen. However, the effect of carbon dioxide becomes blunted once these chemoreceptors develop tolerance to chronically elevated PaCO_2_, which shifts the respiratory drive to being stimulated by hypoxia. When patients with COPD inspire high oxygen content, the resultant decrease in hypoxic drive may be responsible for the worsening retention of PaCO_2_ seen in our study [26,29]. There may be other physiologic effects responsible for the observed increase in PaCO_2_ given that we did not find a statistically significant correlation between the change in PaCO_2_ and respiratory rate. Another potential explanation could be related to patients with obstructive lung disease being uniquely at risk for dynamic hyperinflation, which can occur due to increases in end expiratory lung volumes (a known consequence of HFNC) [5]. Our study also did not directly measure tidal volumes, esophageal pressures to obtain intrinsic PEEP, or the work of breathing. Studies analyzing these effects in COPD patients have demonstrated reductions in respiratory effort leading to reduced work of breathing [28]. Even though we did not examine these responses, the current studies lay the foundation for evaluating these outcomes in patients treated with HFNC.

Our findings suggest that patients with COPD who are not hypercapnic at baseline respond favorably to HFNC (without increases in PaCO_2_) even when they receive higher inspired oxygen than needed, whereas those who are hypercapnic at baseline are at an increased risk for further CO_2_ retention when receiving increased inspired oxygen. Multiple physiologic effects factors contribute to the variable responses to HFNC reported by others [26] which results in a reduction in PaCO_2_ in some subjects, while in others, PaCO_2_ increases in response to HFNC [6,26]. Whereas PaCO_2_ tends to decrease in a flow-dependent manner in COPD patients [20], increased oxygen fraction in baseline hypercapnic patients worsened hypercapnia when flow remained the same, hence careful titration of delivered oxygen is particularly important in these patients. For patients experiencing acute respiratory failure who are treated with NIPPV, many experts recommend careful monitoring after the initiation of NIPPV and to consider checking an arterial blood gas after 1 h of therapy to ensure there is no worsening of ventilation [40]. We believe that a similar protocol should be considered in COPD patients undergoing HFNC due to the multiple physiologic effects at play and the potential for worsening hypercapnia.

We observed that monitoring our patients with transcutaneous capnography throughout the study allowed us to identify patients who had worsening hypercapnia during the study. This suggests that monitoring patients during HFNC, especially those with high FiO_2_, may allow for safe application for this method. We also noted good agreement among our transcutaneous values and arterial blood gas values. However, such an observation was different from that reported by others who did not find a consistent relation between blood gas values and transcutaneous readings [41]. These data suggest that transcutaneous capnography should be used with caution in an acute setting. Our data also suggest that monitoring for worsening CO_2_ retention is essential in patients with COPD who are treated with HFNC with high FiO_2_.

Even though this was an open-label, nonblinded study, our comparisons were done serially in the same patients, which increased the robustness of our observations. However, given that our study followed a sequence of study conditions (from baseline supplementary oxygen to HFNC at baseline FiO_2_ to HFNC at 30% higher FiO_2_), it is possible that the order of study conditions may have affected our results, and future studies may consider randomizing the study conditions to exclude the influence of the sequence. Furthermore, despite a concern about the small number of subjects in each subgroup, the numbers are similar to those reported with previous physiologic studies and are adequately powered based on previous analyses [26]. Patients with neuromuscular disease or obstructive sleep apnea/obesity hypoventilation syndrome were excluded to allow for a homogenous, well-characterized population of COPD with and without hypercapnia. Our study focused on severe COPD cases, with an average FEV1 of 34%. Therefore, we believe that decreased expiratory flow rate was primarily responsible for the physiologic response to HFNC. Of note, we evaluated the acute responses to HFNC with the flow kept constant and oxygen fraction increased, which has not previously been evaluated. We did not assess long-term outcomes as other studies because the adaptive responses are different during acute and long-term studies [22,23,24]. It is also possible that a longer period of treatment may show a different response to HFNC than the acute responses. Since acute responses to altered oxygen therapy are applied frequently during decompensated COPD, our studies are of high clinical relevance in patients with severe COPD who require high flow oxygen. Future studies are needed in the COPD population in order to maximize the physiologic benefits (through nasopharyngeal wash out and decreased respiratory effort/work of breathing) and to minimize the effects of oxygen-induced hypercapnia.

## 5. Conclusions

We show that severe COPD patients with baseline hypercapnia are more likely to develop further CO_2_ retention when increased oxygen fraction was delivered by HFNC and flow remained unchanged. Retention of CO_2_ during HFNC is likely due to loss of hypoxic drive, although other mechanisms, including V/Q mismatch or the Haldane effect, may also mediate such a response in severe COPD patients. Our study complements reports by others and emphasizes the critical need to titrate HFNC to minimize the oxygen fraction delivered and optimize the flow in order to attenuate this potential response of acute worsening hypercapnia in severe COPD patients.

## Figures and Tables

**Table 1:** Clinical characteristics of patients with severe COPD (FEV1 < 50%).

Patient Characteristics	*n* = 11
Age (mean (SD))	66.45 (7.61)
BMI (median [IQR])	25.50 (22.80, 33.55)
Gender (%)	
Males	9 (81.8)
Females	2 (18.2)
FEV1 in L (median (IQR))	0.90 (0.84, 0.97)
FEV1% (mean (SD))	34 (8)
O_2_% (mean (SD))	28 (5)
O_2_ supp (%)	
Exertion	2 (18.2)
2 L	5 (45.5)
3 L	1 (9.1)
4 L	3 (27.3)
pH (mean (SD))	7.37 (0.04)
PaO_2_ (mean (SD))	68.00 (63.50, 84.00)
PaCO_2_ (mean (SD))	49.46 (12.43)
Transcutaneous CO_2_ (mean(SD))	48.04 (11.23)

Normally distributed variables are presented as mean ± standard deviation. Non-normally distributed variables are presented as median ± interquartile range. BMI: body mass index, FEV1: forced expiratory volume, L: liters, PaO_2_: partial pressure of oxygen on baseline supplemental oxygen, Supp: supplemental oxygen in liters, Exertion: supplemental oxygen to be used with exertion, PaCO_2_: partial pressure of carbon dioxide, SD: standard deviation, IQR: interquartile range.
